# Rational design of monomeric IL37 variants guided by stability and dynamical analyses of IL37 dimers

**DOI:** 10.1016/j.csbj.2024.04.037

**Published:** 2024-04-22

**Authors:** Inci Sardag, Zeynep Sevval Duvenci, Serkan Belkaya, Emel Timucin

**Affiliations:** a Bogazici University, Department of Molecular Biology and Genetics, Istanbul 34342, Turkey; b Acibadem Mehmet Ali Aydinlar University, Institute of Health Sciences, Department of Biostatistics and Bioinformatics, Istanbul 34752, Turkey; c Bilkent University, Department of Molecular Biology and Genetics, Ankara 06800, Turkey; d Bilkent University, The National Nanotechnology Research Center (UNAM), Ankara 06800, Turkey; e Acibadem Mehmet Ali Aydinlar University, School of Medicine, Biostatistics and Medical Informatics, Istanbul 34752, Turkey

**Keywords:** IL37, Molecular dynamics simulations, Dimer, Stability, In silico protein engineering

## Abstract

IL37 plays important roles in the regulation of innate immunity and its oligomeric status is critical to these roles. In its monomeric state, IL37 can effectively inhibit the inflammatory response of IL18 by binding to IL18R*α*, a capacity lost in its dimeric form, underlining the pivotal role of the oligomeric status of IL37 in its anti-inflammatory action. Until now, two IL37 dimer structures have been deposited in PDB, reflecting a substantial difference in their dimer interfaces. Given this discrepancy, we analyzed the PDB structures of the IL37 dimer (PDB IDs: 6ncu, 5hn1) along with a AF2-multimer prediction by molecular dynamics (MD) simulations. Results showed that the 5hn1 and AF2-predicted dimers have the same interface and stably maintained their conformations throughout simulations, while the recent IL37 dimer (PDB ID: 6ncu) with a different interface did not, proposing a possible issue with the recent IL37 dimer structure (6ncu). Next, focusing on the stable dimer structures, we have identified five critical positions of V71/Y85/I86/E89/S114, three new positions compared to the literature, that would reduce dimer stability without affecting the monomer structure. Two quintuple mutants were tested by MD simulations and showed partial or complete dissociation of the dimer. Overall, the insights gained from this study reinforce the validity of the 5hn1 and AF2 multimer structures, while also advancing our understanding of the IL37 dimer interface through the generation of monomer-locked IL37 variants.

## Graphical abstract

**Figure:**
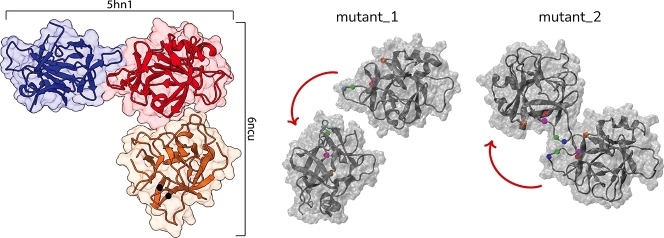

## 1 Introduction

Members of the interleukin-1 (IL1) family are involved in the regulation of innate immunity and inflammation [1]. Although this cytokine family is primarily associated with pro-inflammatory functions, certain members have been identified to play anti-inflammatory roles [2], [3]. IL37 stands out among the anti-inflammatory members of the IL1 family because it competes with IL18 to bind to the IL18 receptor *α* (IL18R*α*) [1]. Binding of IL37 to IL18R*α* recruits a different co-receptor named IL1R8 (SIGIRR) to the binary complex rather than IL18R*β*, leading to blockage of the inflammatory response of IL18 [4], [5]. Given these critical functions, IL37 has been recognized as a fundamental factor in the regulation of innate immunity [6].

IL37 can form a homodimer structure [6], which has been shown to attenuate its anti-inflammatory effect, probably through a steric effect blocking its interaction with IL18R*α* [7]. In other words, the monomeric form of IL37 exhibits greater efficacy in inhibiting inflammation compared with the dimeric form [6]. This perspective consolidates the importance of the dimer structure and dimer interface of IL37 for the design of IL37-based therapeutics. The current literature on the IL37 dimer interface is well established, with multiple studies strongly supporting the validity of a symmetric interface lined by residues D73, K83, and Y85 [5], [6], [8], [9].

There are two IL37 structures in PDB and both were annotated as homodimers. The first structure (PDB ID: 5hn1) covers residues 46 to 218 [6], while the recent structure (PDB ID: 6ncu) covers a slightly more compact region that includes residues 53 to 206 [5]. IL37 in both PDB structures has a *β*-trefoil fold with 12 *β*-strands and 3 *α*-helices [6]. Despite well-established knowledge of the IL37 dimer interface [5], [6], [8], [9], the 6ncu structure does not form the well-recognized head-to-head dimer, instead, it has a dimer interface formed by asymmetric regions of IL37 subunits.

IL37 engineering strategies aimed at developing variants with decreased dimerization potential and increased monomer stability hold promise as potential avenues for designing IL37-based anti-inflammatory therapeutics [6]. Three key mutations, namely Y85A and D73K/A, have been identified as effective in inhibiting inflammation [8], attributed to their capacity to disrupt the dimeric form [5], [6]. However, these monomer-locked variants have been reported to have limited therapeutic applications because of their small size and short half-lives [8], [10]. Furthermore, two of these variations are alanine substitutions, which is arguably perceived as the best substitution to test the functional impact of a mutation [11], [12], [13]. However, systematic investigation of large-scale mutagenesis libraries addressed that charged or polar amino acids are often better at disrupting intermolecular interactions than alanine [14]. Thus, we noted that the potential of these critical positions has not been fully explored, reflecting the possibility of a better substitution than alanine for the Y85 and D73 positions. Overall, recognizing the importance of the IL37 dimer and monomer structures in the design of anti-inflammatory therapies [5], [6], we utilized a computational design approach to assess the impact of site-saturation mutagenesis on the stability of both monomer and dimer structures of IL37.

Building on the success of computational methods in revealing the structural basis of inflammation in other IL1 family members [15], this study utilizes both molecular dynamics (MD) simulations and AF2-multimer calculations to shed light on the structure and dynamics of the IL37 dimer. Our study extensively investigated the IL37 dimer structures using MD simulations. We observed that the 6ncu dimer structure was not stable in MD simulations and repeatedly underwent immediate disruption of the crystal interface. Furthermore, the AF2-multimer predicted IL37 dimer adopted the identical interface with the 5hn1 homodimer. Through extensive analysis of the 5hn1 dimer, we have identified promising positions of IL37 that would destabilize the dimer structure, preserving the stability of monomers. We then led the rational design of two monomer-locked variants of IL37 and computationally validated the stability of mutant monomers and dimers. All together, we reported (i) critical issues with respect to one of the PDB structures of the IL37 dimer, reflecting the capacity of computational methods and predictions to identify the correct protein-protein interfaces and (ii) two quintuple variants to contribute to the development of stable monomer-locked IL37-based therapeutics.

## 2 Methods

### 2.1 Dimer structures

IL37 homodimer structures with the IDs of 6ncu and 5hn1 were extracted from the PDB. Non-terminal missing regions in both dimers were modeled by the MODELLER [16]. Several loop models were generated and the final models were selected according to the lowest normalized discrete optimized protein energy score (zDOPE), for which a negative score indicates better predictions [17]. The homodimer structure of IL37 was also predicted by the AlphaFold2 (AF2) multimer [18], [19], [20], [21]. MSAs were performed with the MMseqs2 software [22], [23] without template mode. MSA mode was selected as MMseqsUniRef + Environmental, while pair sequences of the same species and unpaired MSA were chosen as the pairing mode [24]. The number of cycles was adjusted to 48. The predicted structure was relaxed by the AMBER force field [25]. The final model was selected based on the confidence score (pLDDT), which relies on the lDDT-C*α* metric [26].

### 2.2 Molecular dynamics simulations

Three IL37 dimer structures composed of two PDB (6ncu and 5hn1) and an AF2-computed structure were analyzed using MD simulations. All MD systems were prepared using CHARMM-GUI [27], [28], [29]. Crystal water and other solvent molecules were removed and the structures were protonated according to pH 7.0 and 2.5 using the PDB2PQR tool [30], [31]. The complexes were placed in the center of rectangular boxes with 10 Å of edge distances, which were fit according to the size of the complexes. Na^+^ and Cl^−^ counterions were placed to neutralize the systems to a final concentration of 0.15 M. MD simulations were carried out using the NAMD engine [32] and the CHARMM36m force field with a *ϕ*, *ψ* grid-based energy correction map [28], [33], [34]. Water molecules were explicitly treated by the TIP3P model [35]. Periodic boundary conditions and a time step of 2 fs were applied to all simulations. The particle mesh Ewald method was applied to calculate long-range electrostatic interactions with a grid spacing of 1 Å [36]. A cutoff distance of 12 Å was used for the non-bonded interaction terms. Three IL37 homodimer systems were energy-minimized in 10,000 steps and equilibrated for 250 ps in an *nVT* ensemble. Finally, the systems were simulated under constant pressure (1 atm) and temperature (310.15 K) using the *nPT* ensemble using the Langevin thermostat and piston pressure method [37], [38], [39]. Production simulations were repeated three times for each dimer structure.

### 2.3 Trajectory analysis

Production MD trajectories were analyzed by means of pairwise root mean square displacement (RMSD) and fluctuation (RMSF) of C*α* atoms. For the former, MDanalysis scripts were used [40], [41]. A time-dependent change in the solvent-accessible surface area (SASA) of the dimer and its subunits was monitored. SASA at the interface was calculated as the difference in the SASA measurement between the sum of each subunit and the dimer. Visualization of structures and trajectories was performed using Visual Molecular Dynamics (VMD) [42]. UCSF ChimeraX tool was also used to track visualization static structures [43], [44], [45]. Bio3D package was used for the principal component analysis (PCA) [46]. SASA and fluctuation calculations were performed using the command measure of the VMD [42].

### 2.4 Foldx calculations

Two snapshots from each replicate trajectory were selected, one from the last 10 ns of the first half of the trajectory and the second from the last 10 ns of the second half. Each system was represented by a total of seven snapshots, including the static structure (PDB or AF2 computed structure). Each snapshot was then analyzed using the protein design tool, FoldX, which leverages an empirical force field to predict the folding/binding free energy of a protein structure/complex [47]. FoldX force field considers various factors that influence protein structure, including Van der Waals, Coulomb, hydrogen bond, solvation, and torsional energy terms. The FoldX command AnalyseComplex was initially run to gather the amino acids at the interface for the 5hn1 structure. This list of interface amino acids was then analyzed using the Pssm command to calculate their contribution to the binding free energy of the dimer. Site-saturation mutagenesis was considered for each selected position. In addition, the command AlaScan was used to map the contribution of IL37 amino acids to the folding free energy of the dimers and subunits.

### 2.5 MM-PBSA calculations

Relative binding free energy calculations of the IL37 homodimers were performed using the MM-PBSA module [48] of AmberTools [49]. $\Delta G_{\mathrm{dimer}}$ is calculated using the formula,

(1) $$\Delta G_{\mathrm{dimer}}=\Delta E_{MM}+\Delta G_{solv}$$

wherein, the vacuum energy, $\Delta E_{MM}$ is composed of van der Waals and Coulomb energy terms, while the solvation energy, $\Delta G_{solv}$ is composed of $\Delta G_{pol}$ and $\Delta G_{nonpol}$ terms. The internal dielectric constant was set at 4 [50], [51] and the grid space was set to 2.0 Å. For each dimer including the quintuple mutants, the calculations were performed on 25 different snapshots that were extracted from the first and last parts of the trajectories.

## 3 Results

### 3.1 Static structures of human IL37 dimer

**Fig. 1:**
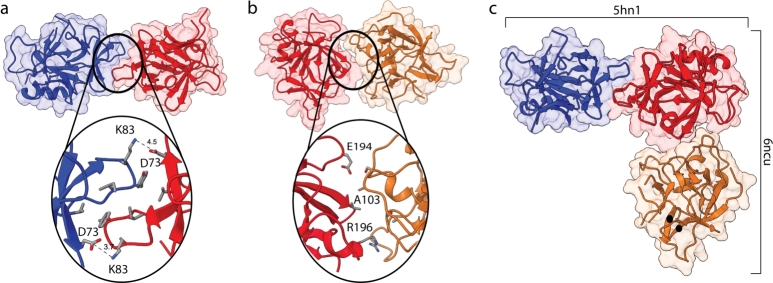
Binding interface of two crystal dimers 5hn1 and 6ncu from PDB. (a) Hydrophobic interface of the 5hn1 dimer composed of V71, Y85 and I86 from both subunits, which is sectioned by two symmetrical intermolecular interactions between K83 and D73, is visualized. (b) The interaction surface of the 6ncu is depicted showing the amino acids that are closely found at the interface. (c) Best superimposition of two biological assemblies were shown based on C*α* RMSD.

IL37 dimer was captured in two crystal structures (PDB IDs: 6ncu and 5hn1) [5], [6]. Fig. 1a-b depicts the biological assemblies of these structures. A cyclic C2 symmetry was described for the biological assembly of 5hn1 (Fig. 1a) [6], which refers to a rotation of 180^∘^ around a particular line. However, no recognizable symmetry elements were found for the 6ncu structure (Fig. 1b) [5]. Close-up views of the dimer interfaces also reveal the conflict in the dimer configurations of two assemblies. Specifically, the 5hn1 dimer interface was formed by a symmetrical contour, whereas it was not the case for the 6ncu dimer. Two symmetric electrostatic interactions between K83-D73 link two subunits at the poles of the binding interface of 5hn1 (Fig. 1a), leaving a hydrophobic cluster in the interior. However, neither the biological assembly nor the asymmetric unit of 6ncu structure shared the same interface with the 5hn1 dimer (Fig. 1b). Instead, the 6ncu dimer interface contoured the asymmetric surfaces of the IL37 monomers, resulting in a less compact interface without any detectable network of interactions (Table S1). The superimposition of both dimers further confirmed the difference in the biological assemblies of these two structures (Figs. 1c and S2). Superimposition of these dimers relies on only chain A and chain B, reflecting that the 6ncu dimer interface is asymmetrically formed by distinct regions of the subunits (Fig. S2). Furthermore, the interface reported in the 6ncu dimer was quite different from that of the 5hn1 dimer (Figs. 1 and S3). In general, this comparative analysis of two biological assemblies from PDB implies a deviation of the interface of 6ncu from the dimer interface of IL37 previously identified in the literature [6].

The IL37 homodimer was also predicted by the AF2 multimer (Fig. S1), showing a highly accurate prediction based on pLDDT and predicted aligned error (PAE) scores. The superposition of the 5hn1 and AF2 dimer structures in (Fig. S1) showed that the AF2 model adapted the same dimer configuration with the 5hn1 structure with an average change in C*α* RMSD of 1.3 Å. The general structures and binding interfaces of the AF2 and 5hn1 dimers were in close agreement, except for two disordered regions exhibiting particularly low pLDDT scores, and thus contributing to a slight increase in RMSD. We also calculated the surface area of the dimer interfaces in these structures (Table S1). In particular, the area of the 6ncu dimer interface was smaller than the 5hn1 and AF2 dimers. Detailed analysis of the static dimer structures revealed an apparent discrepancy in the PDB structures of 6ncu and 5hn1 as well as in the AF2 computed dimer.

### 3.2 Dimer dynamics and stability

After modeling the missing regions of the PDB structures, we analyzed all three IL37 dimers by MD simulations of all atoms. Details of the MD systems are given in Table S2. All simulations lasted for 0.5 μs and were repeated three times. Pairwise C*α* RMSD was calculated for all dimers (Fig. S4-dimer). The dimers 5hn1 and AF2 did not show significant C*α* mobility when the dimer backbone was set as the reference, while the 6ncu dimer backbone has reached RMSD values as high as 20 Å for all three simulations (Fig. S4-dimer). To identify whether this large mobility of 6ncu is due to monomer or dimer instability, we extended the RMSD calculations using only a single monomer structure as the reference. For all three dimers, chain A remained intact throughout all simulations (Fig. S4-chainA). For the 5hn1 and AF2 dimer structures, a slight increase in the C*α* mobility of chain B was reported with respect to chain A, the observation that was expected given the reference alignment on chain A (Fig. S4-chainB). However, the 6ncu dimer showed a highly mobile chain B with respect to chain A for all repeated simulations (Fig. S4-chainB), suggesting a disruption of the dimer structure. We also extracted essential dynamics of each system by cartesian PCA (Fig. S5). Score plots obtained from the first three principal components (PCs) corroborated that the 6ncu dimer spanned a conformational space much larger than that of the other dimers (Fig. S5). Together, these results unraveled the instability of the 6ncu dimer, while for the 5hn1 and AF2 structures, neither dimer nor monomers showed an abnormal level of backbone mobility that could be perceived an indication of instability.

**Fig. 2:**
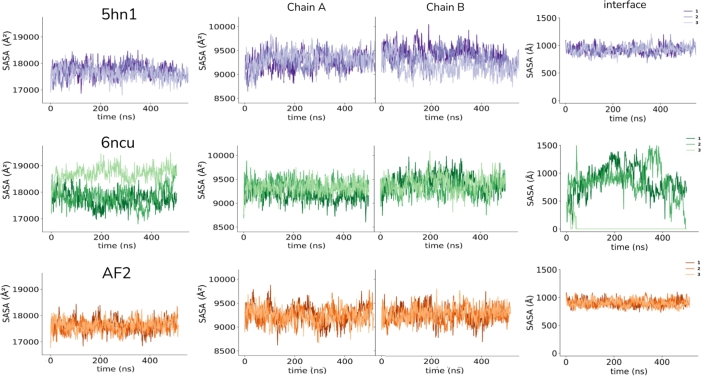
Solvent accessible surface area (SASA) changes were calculated for all three dimers. First column shows the dimer SASA, the second and third columns show monomer SASA values, and the last column shows the interface SASA calculated by subtracting the dimer SASA from the sum of subunit SASA values.

We also examined the solvent-accessible surface area (SASA) of the dimer, monomers, and interface of the three systems (Fig. 2). The SASA of the dimers and monomers did not significantly change for the 5hn1 and AF2 dimers. However, in the 6ncu structure, the dimer and the interface SASA largely fluctuated. In particular, the interface SASA was measured to be zero for one of the 6ncu simulations (Fig. 2, lightest green), indicating complete dissociation of subunits. However, the 5hn1 and AF2 dimers that share the same interface demonstrated a stable dimer interface, as evidenced by flat SASA measurements of the interface (Fig. 2).

**Fig. 3:**
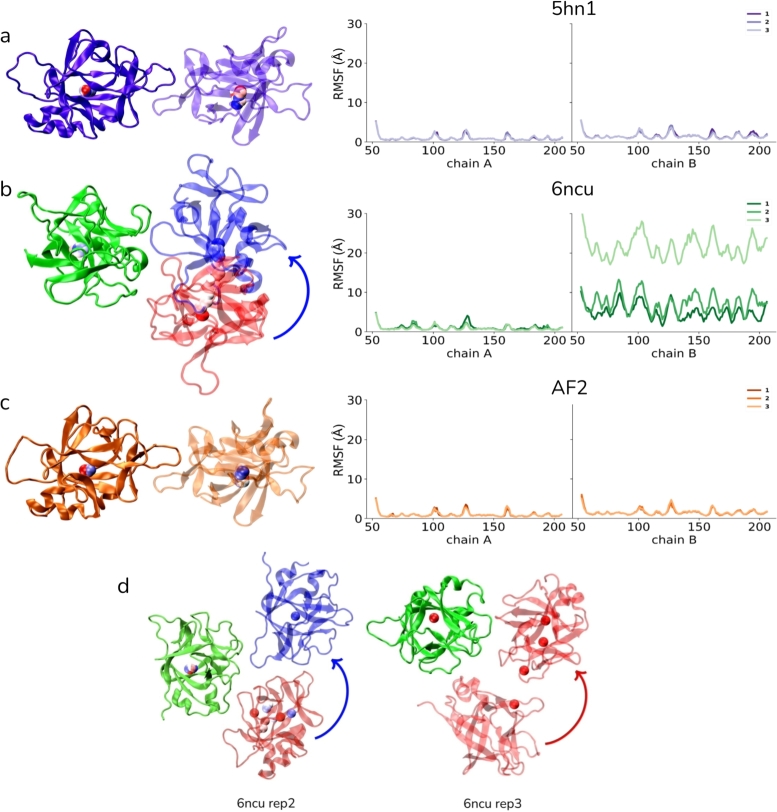
The relative displacement of one monomer (chain B) with respect to the other (chain A) for the first simulations of the (a) 5hn1, (b) 6ncu and (c) AF2 dimers. For each visual, center of mass of the monomers were shown by spheres colored according to simulation time (red: start, blue: end). (d) Panel shows the rest of replicate simulations of the 6ncu, wherein the dimer subunits are almost completely dissociated.

To further investigate how the overall structure of the dimer has changed, a reduced trajectory of the first simulations was visualized for each system (Fig. 3). These reduced trajectories show the initial and final structures of chain B of the 6ncu in red and blue, respectively, just before complete separation and also show the shortened trajectory of the center of mass of each subunit using a sphere representation. Given this analysis, there is almost no relative change in the orientation of the subunits in the 5hn1 and AF2 dimers. However, the 6ncu dimer showed a dramatic movement of chain B with respect to chain A, indicating the disruption of the dimer conformation reported in the crystal structure.

The right panel in Fig. 3 shows C*α* fluctuations for each monomer, where no major fluctuations were observed for the 5hn1 and AF2 dimers. On the other hand, C*α* fluctuations in monomers were observed in all simulations of the 6ncu. However, all residues in one of the 6ncu dimer monomers showed fluctuations much higher than those in the other monomer, indicating that one of the 6ncu monomers has been displaced remarkably with respect to the other chain (Fig. 3b,d). (See supplementary movies - 5hn1 (purple), 6ncu (green)).

**Fig. 4:**
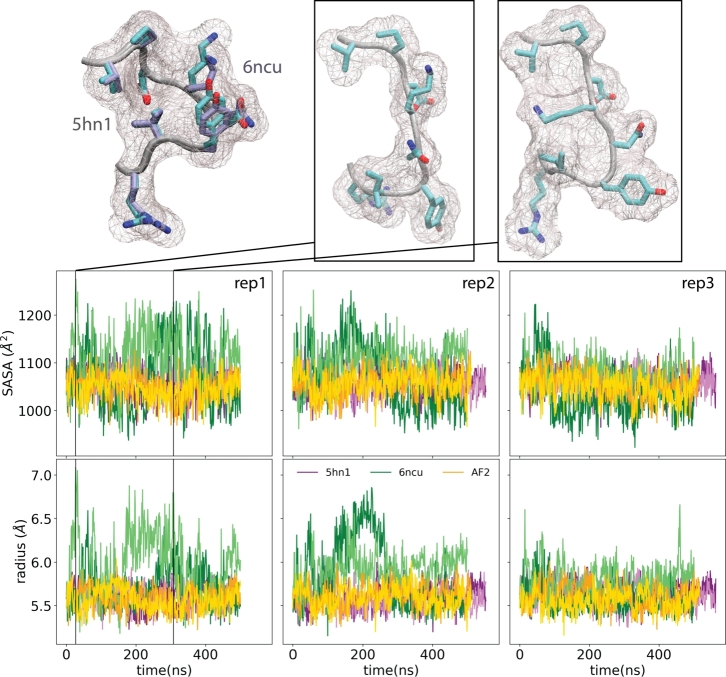
Superimposed dimer interface (80-87) of 5hn1 (mauve) and 6ncu (cyan) structures were shown. SASA and radius of gyration (*R*_*G*_) of this interface were measured throughout simulations for both chains. 6ncu chains were colored by green, 5hn1 chains were colored by orange and AF2 chains were colored by purple. Top row contains two representative snapshots from the 6ncu simulations showing a notable change in the SASA and *R*_*G*_ of the epitope during simulations.

We also traced the SASA of each subunit interface in all systems (Fig. 4). First, we note that the PDB conformations of the subunit interfaces formed by the continuous epitope between residues 80-87 were perfectly aligned with each other in two crystal structures, 5hn1 and 6ncu. However, during the simulations, this surface epitope in the 6ncu subunits underwent notable changes that resulted in an extension of this surface, disrupting the hydrophobic cluster mediated by Y85. On the other hand, two other structures, 5hn1 and AF2-computed dimers, did not show any apparent change in shape or SASA of this continuous epitope that forms the dimer interface. This analysis particularly pointed out that the subunit interface between 80-87 was highly flexible in the 6ncu dimer, undergoing conformational changes that would limit this epitope's interaction with the other subunit.

We conducted an *in silico* alanine scanning experiment by FoldX for all three IL37 dimer structures (Fig. S6). A selected set of seven snapshots including the PDB structure and two snapshots from each repeated simulation were recruited to calculate the average change in stability (ΔΔ*G*) after the alanine mutation of each position in the IL37 dimer. Essentially, alanine substitution at the dimer interface positions of the 6ncu dimer resulted in negligible changes in stability. On the other hand, alanine substitutions at the dimer interface of the 5hn1 and AF2 structures destabilized the structure by more than 2 kcal.mol^−1^. This analysis further revealed the presence of additional interactions in the 5hn1 and AF2 dimers, which were absent in the 6ncu dimer. In general, the extensive analysis of all-atom MD simulations underscored that among the two distinct dimer conformations reported in the PDB, only 5hn1 is a stable homodimer.

We also estimated the binding free energy ($\Delta \Delta G_{\mathrm{dimer}}$) of the dimers with the molecular mechanics Poisson-Boltzmann surface area (MM-PBSA) method [50], [51] in addition to FoldX [47] calculations (Table 1). Both techniques indicated that the IL37 dimers from the 5hn1 and AF2 structures maintained a stable conformation during the simulations, whereas the 6ncu dimer did not.

**Table 1:** Dimer stability analysis.

	FoldX^†^	MM-PBSA^‡^	
		first	last
5hn1 (WT)	-5.34	-3.23±0.55	-3.77±0.71
AF2 (WT)	-6.13	-4.63±0.75	-3.36±0.41
6ncu (WT)	1.20	6.25±0.62	10.29±0.31

V71R/Y85C/I86W/E89L/S114R	6.52 (0.76)	7.84±0.55	10.41±0.56
V71R/Y85K/I86W/E89L/S114R	8.43 (3.21)	10.39±0.58	9.43±0.58

^†^ Change in monomer stability is given in parentheses.

^‡^ First and last parts of the simulations were used (Fig. S7).

To mimic the subphysiological crystallization condition of the 6ncu structure [5], we protonated both dimers (5hn1 and 6ncu) according to pH 2.5 and similarly simulated them in MD simulations. The resulting dimers had most of the ionizable residues (D, E and H) in the protonated state (Table S3). Analysis of the trajectories showed that the dimer dynamics was not affected by the change of the protonation state of the listed amino acids in Table S3. Both systems displayed dynamics similar to those of the unprotonated states. The 5hn1 dimer maintained its crystal dimer conformations stably, while 6ncu did not (see suplementary movie). Although crystallization conditions were not completely replicated in these additional simulations, e.g. we did not incorporate 10% MPD, the dimer dynamics were not affected by protonation of acidic amino acids in either dimer.

### 3.3 Human IL37 variants and their impact on stability

**Fig. 5:**
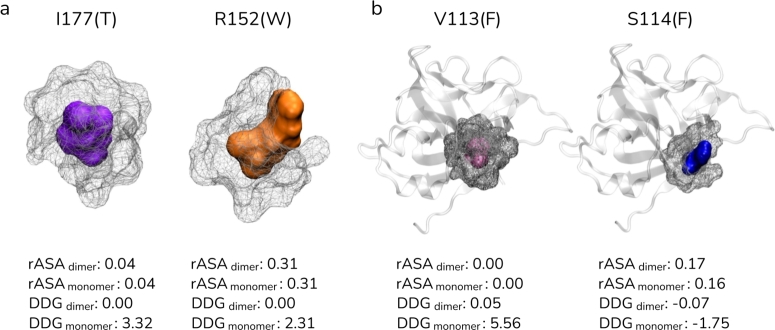
The locations of selected human IL37 variants, I177T, R152W, V113F and S114F and other residues within range 5 Å. (a) The positions I177 and R152 and (b) V113 and S114 along with surrounding amino acids were shown in surface representations. Relative accessible surface area for the dimer and monomer structures were also given for all amino acids. The impact of the observed human variants of these positions on the dimer and monomer stability were given in kcal.mol^−1^.

We have next extracted all reported human missense variations of mature IL37 (ENST00000263326.8) from gnomAD v3.1.2 [52] and investigated their impact on the monomer and dimer stability by FoldX [47] (Table S4). Only two of these variants, I177T and R152W, were associated with a clinical significance label of pathogenic and benign, respectively. Essentially, these two positions did not make any close contact with the other subunit; i.e., they are not found at the dimer interface. In line with this, the substitutions of I177T and R152W did not affect the stability of the $\Delta \Delta G_{\mathrm{dimer}}$ (Fig. 5a). However, both substitutions caused a destabilization impact on the monomer structure, with a positive $\Delta \Delta G_{monomer}$ score greater than 2 kcal.mol^−1^. Although I177T and R152W had a similar magnitude of destabilization effect, I177T was pathogenic and associated with inflammatory bowel disease [53], while R152W was a benign variant according to ClinVar. This discrepancy could have been due to the relative accessibility of the positions in question. In particular, I177T was located at the core and its side chain was not accessible. On the other hand, R152 was not completely buried and was much more accessible than I177 (Fig. 5a). Therefore, the substitution of R152 to another bulky amino acid of W would impact the core structure of IL37 less than the substitution of I177T would.

We reported a similar case for two other substitutions V113F and S114F, that FoldX calculations led to contradictory scores although these substitutions are adjacent positions and substituted for the same amino acid (Fig. 5b). In particular, V113F led to a notable decrease in monomer stability by 5.6 kcal.mol^−1^, while S114F led to a slight increase by -1.8 kcal.mol^−1^ (Fig. 5b). This large difference could be related to the higher accessibility of S114 compared to V113. V133F, albeit being a complementary mutation, V-to-F, would disrupt the tight interactions formed by V113. Therefore, V113F may be an important variation given its parallel outcome with clinically significant I177T.

### 3.4 Design of novel IL37 variants with reduced dimer stability

Seven snapshots of 5hn1 and AF2 that were obtained from repeated MD simulations were used. We carried out *in silico* site-saturation mutagenesis for each selected position and analyzed their impact on the stability of the dimer (Fig. S8) and monomer forms (Fig. S9). We pursued substitutions that yielded positive scores in the binding free energy of the dimer ($\Delta \Delta G_{\mathrm{dimer}}$), which is an indication of dimer destabilization (Fig. S8) and negative scores in the $\Delta \Delta G_{monomer}$, as an indication of monomer stabilization (Fig. S9).

Any substitutions at the positions of Y85 and I86 showed a dimer destabilization of more than 1 kcal.mol^−1^ (Fig. S8), confirming the pivotal involvement of these positions in dimerization [54]. By considering both Fig. S8 and Fig. S9, we have reported three additional positions of V71, E89, and S114 that would alter the dimer structure without significantly affecting the monomer stability. In general, the V71R, Y85C/K, and I86W mutations were selected as the most promising dimer destabilizing mutations (Fig. S8) with a slight destabilizing effect on monomers (Fig. S9). Furthermore, the E89L and S114R mutations were chosen due to their stabilizing impact on the monomer structure (Fig. S9). In general, two quintuple mutants were generated that included the mutations V71R/Y85C/I86W/E89L/S114R and V71R/Y85K/I86W/E89L/S114R and varied only for the substitution of Y85. We pursued both a cysteine and a lysine substitution at the Y85 position, as the FoldX analyzes underscored a large dimer destabilizing impact for both substitutions (Fig. S8). Furthermore, Y85C is one of the human variants reported that has a high $\Delta \Delta G_{\mathrm{dimer}}$ value according to FoldX (Table S4). In particular, Y85C destabilized the dimer structure by 2.91 kcal.mol^−1^ and the monomer by 0.92 kcal.mol^−1^. Similarly, Y85K destabilized the dimer structure by 2.38 kcal.mol^−1^ and the monomers by 0.54 kcal.mol^−1^. Quintuple mutants with the only difference between substitution of Y85 to C or K have destabilized the dimer by 6.52 and 8.43 kcal.mol^−1^, respectively. Overall, these analyses showed a pronounced dimer destabilization impact for the quintuple mutations, showing that the destabilization effects of the single mutations were accumulated rather than quenched.

**Fig. 6:**
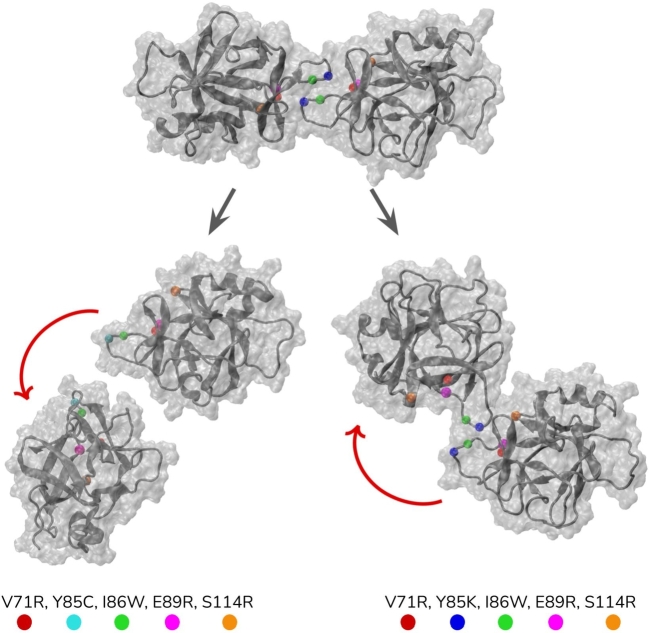
Initial dimer conformation was illustrated at the top. Snapshots that were taken after 29.0 and 50.8 ns from Y85C and Y85K containing mutant simulations, respectively were shown at the bottom. Red arrows indicate the relative movement of one of the monomers with respect to its initial position in the dimer conformation shown at the top.

We generated these quintuple variants using the 5hn1 dimer structure and simulated them similarly in MD simulations. The RMSD plots of mutants showed that both mutants lost the dimer interface shortly after the start of the simulations, resulting in a significant increase in the RMSD values (Fig. S7). Specifically, the mutant containing Y85C led to complete dissociation of the dimer after 29 ns, while the mutant containing Y85K showed significant, although not complete, dissociation of the dimer in Fig. 6 (See supplementary movies). For the latter case, we observed an intermolecular interaction between K85 and D73, in part explaining the partial dissociation of the K85 mutant. We calculated the relative change in dimer stability using FoldX- and MM-PBSA based binding free energy predictions (Table 1). In particular, MM-PBSA calculations were performed for the first and last parts of the trajectory separately labeled in Fig. S7 due to the complete disassociation of one of the mutants after a short while. Both calculations agreed with each other, showing that the dimer structures in the quintuple mutants were largely destabilized (Table 1).

We additionally examined the monomer stability of the quintuple variants. The mutant containing Y85C destabilized the dimer structure without significantly affecting the stability of the monomer, having optimal stability values for the dimer and monomer structures of IL37 (Table 1). However, the mutant that held the Y85K substitution showed a greater monomer destabilization compared to the variant containing Y85C (Table 1). Therefore, the quintuple mutation that holds Y85C is proposed to be a favorable variant that effectively disrupts the dimer structure without affecting the stability of the monomer.

## 4 Discussion

Protein dimerization, a crucial regulatory process impacting the biological function of proteins [55], [56], can occur through various arrangements. Among these, head-to-head dimerization, where the N-termini of each subunit are closely juxtaposed [57], is common among IL1 protein family members [6]. Essentially, IL37 effectively inhibits the inflammatory response led by IL18, a function lost in its dimeric form [6]. Hence, IL37 engineering approaches that create stable monomeric variants of IL37 are recognized as a potential avenue for anti-inflammatory therapies [6]. This recognition underscores the importance of the dimer structure of IL37 that would shape the design process.

The IL37 structures deposited in PDB were annotated as homodimers, albeit they have different dimer conformations (Fig. 1). The biological assembly of 6ncu was assigned by the PDB submitter, while both authors and the Protein Interfaces, Surfaces and Assemblies (PISA), which offers predicted oligomeric structures derived from thermodynamic calculations of complex stability, [58] determined the biological assembly of the 5hn1 structure. For 5hn1, gel filtration and light scattering experiments were used to determine the biological assembly while for the 6ncu, only gel filtration results was reported [5], [6]. Furthermore, these crystals have formed in different space groups, and the 6ncu has a relatively low resolution than the 5hn1. Although the linked publication of 6ncu reports that their structure shares the identical interface with the 5hn1 structure, the PDB coordinates of 6ncu result in a significantly different dimer conformation than 5hn1 (Fig. 1). In line with the notable difference in their dimer interfaces, there are also differences in the dynamics of these dimers (see supplementary movies). In particular, the recent structure (6ncu), which did not have any symmetry elements but formed through an asymmetrically countered dimer interface, was not stable and consistently dissociated, either partially or completely, in all simulations. On the contrary, the first PDB structure (5hn1) and the AF2-computed dimer with a highly similar dimer interface remained intact without any peaked mobility or flexibility that could suggest dimer instability (Fig. 3). An additional noteworthy observation with respect to the static dimer interfaces is the complete inaccessibility of the residues at the 5hn1 dimer interface to the solvent. These residues form airtight interactions with the other subunit, as illustrated in Figure (Fig. 1c and Fig. S3). However, the interface of 6ncu is porous, which would allow water molecules to penetrate (Fig. 1c). This disparity in the compactness of the dimer interfaces contributes to a stability difference in the dimers [59], which partially explained the instability of 6ncu.

The disparities noted in the static and dynamic structures of the dimers could stem from several factors. Annotation problems in the 6ncu structure or inaccuracies in the deposition of its coordinates may account for these discrepancies. Otherwise, the dimer structures used in the simulations were not extensively manipulated, which may result in suboptimal results. The manipulations of the PDB structures were (i) the modeling of missing regions and (ii) removal of crystal water and ligand molecules. First, two very short sections (3, 1 amino acid long) were modeled for the 6ncu structure, while the missing and modeled regions in the 5hn1 structure were relatively longer than those in the 6ncu, noting a similar treatment for both PDB dimers before simulations. Second, the crystal structure of 5hn1 was captured in the presence of 158 water molecules and 5 sulfate molecules [6], while there were no crystal waters and ligands in the 6ncu structure. Therefore, the 6ncu structure did not experience removal of crystal waters or ligands, which may have an effect on the simulation result [60], [61].

The number of water molecules is affected by the resolution of the data. According to Wlodawer et al. at low resolution (∼2.5 Å), it should be possible to discern a maximum of 0.3–0.5 ordered water molecules per protein residue in the electron-density maps, while this number rises to 2 per residue at high resolution (1.0 Å) [62]. Chruszcz et al. also revealed that proteins crystallizing in lower symmetry typically have lower solvent content than those crystallizing in higher symmetry systems [63]. Given the absence of symmetry elements and a resolution of 3.5 Å in the 6ncu structure, it may be reasonable not to identify any water molecules within 6ncu.

Misannotation of the biological unit is an important source of error in the PDB structures [64], [65], [66]. The error rate in the biological unit annotations was estimated to be in the range of 7-15% [65], [67]. These errors can be due to various factors, such as biases during model building, poor resolution, crystallization conditions, and/or biological complexity [64], [65]. Moreover, Xu et al. have pointed out instances [68] where the biological assembly annotation of the PDB structure deviates from the description provided in the associated publication [64]. The situation with 6ncu differs from these examples because its publication [5] also depicted a dimeric state as the biological assembly, consistent with the biological assembly annotation of PDB structure. However, the dimer interfaces described in the publication and the deposited structure do not align.

The binding free energy calculations by Foldx and MM-PBSA converged that the dimers of 5hn1 and AF2 maintained stable dimer conformations throughout the simulations while the 6ncu dimer showed a highly unstable conformation (Table 1). FoldX uses an empirical force field to predict protein stability [47]. The key advantage of FoldX lies in its speed and efficiency. Compared to complex physics-based simulations, it offers a faster and more computationally efficient way to assess protein stability. FoldX stability calculations are often experimentally validated [69], making it a reliable tool for protein stability analysis. We conducted further analysis of dimer stability using the MM-PBSA method, which integrates molecular mechanics energies with Poisson-Boltzmann surface area continuum solvation [50], [51], [70]. Overall, our computational analyses validated the 5hn1 and AF2 structures as the stable dimer, and noted particular inconsistencies for the dimer structure of 6ncu.

While D73 has been previously identified as an effective amino acid situated at the dimer interface, forming a salt-bridge interaction with K83 in the alternate subunit [6], our *in-silico* stability analysis revealed that substitutions at either D73 or K83 were not pivotal for dimer stability (Fig. S8). Instead of these charged interactions, our analysis emphasized the significance of the hydrophobic cluster at the dimer interface for both dimer and monomer integrity. Specifically, destabilization of this cluster comprising V71, Y85, I86, and particularly the hydrocarbon side chain of K83 (Fig. 1a), not only compromises the dimer interface (Fig. S8) but also disrupts intramolecular interactions of the monomer (Fig. S9). Therefore, any substitution at these positions should be made cautiously, even though they are intuitively prime candidates for the disruption of the IL37 dimer due to their close location to the interface.

After conducting a thorough analysis of seven distinct snapshots obtained from three independent MD simulations, we pinpointed the positions of V71, Y85, I86, E89, and S114 where substitutions would destabilize the dimer while stabilizing or neutralizing the monomer. Y85 emerged as a key player in the hydrophobic cluster crucial for both dimer and monomer stability, as evidenced by the monomer-locked status of Y85A [5]. Our findings indicated that substitutions with cysteine or lysine were more effective at destabilizing the dimer at the Y85^th^ position compared to alanine substitution. Moreover, a comparison between the Y85C and Y85K variants highlighted Y85C, a reported human variant, as the most optimal substitution due to its ability to prevent an additional interaction with D73 in the opposing monomer, which was otherwise observed in the Y85K containing mutant (Fig. 6). Ultimately, the quintuple mutation of V71R/Y85C/I86W/E89L/S114R were shown to be effective in destabilizing the dimer while preserving monomer structure (Figs. 6, S8-9).

In summary, we (i) demonstrate that the correct IL37 homodimer in the PDB is the 5hn1 structure, whereas the dimer interface depicted in the 6ncu structure is unstable in simulations, with particular emphasis on the merits of computational approaches in analyzing complex structures; (ii) propose a rational design involving five mutations in IL37 to modulate both dimer and monomer stability.

## CRediT authorship contribution statement

**Inci Sardag:** Data curation, Formal analysis, Visualization, Writing – original draft, Writing – review & editing. **Zeynep Sevval Duvenci:** Data curation, Formal analysis, Visualization, Writing – original draft, Writing – review & editing. **Serkan Belkaya:** Conceptualization, Funding acquisition, Writing – review & editing. **Emel Timucin:** Conceptualization, Project administration, Supervision, Writing – original draft, Writing – review & editing.

## Declaration of Competing Interest

The authors declare that they have no known competing financial interests or personal relationships that could have appeared to influence the work reported in this paper.

## Appendix A Supplementary material

MMC 1 Supplementary file containing 4 Tables and 9 Figures about details of IL37 structures, MD systems, FoldX and molecular dynamics simulations. MMC 1

MMC 2 Human variations of IL37. MMC 2

Video 1 Movie showing the MD trajectories of 5hn1 dimer structure. Video 1

Video 2 Movie showing the MD trajectories of 6ncu dimer structure. Video 2

Video 3 Movie showing the MD trajectories of the quintuple mutant dimers. Video 3

Video 4 Movie showing the MD trajectories of 5hn1 and 6ncu dimers at pH 2.5. Video 4
