# Supplementary material for: Rational design of monomeric IL37 variants guided by stability and dynamical analyses of IL37 dimers
Source: Comput Struct Biotechnol J. 2024 Apr 22;23:1854–63. doi: 10.1016/j.csbj.2024.04.037 (PMC11177541; doi:10.1016/j.csbj.2024.04.037)
Supplement: MMC 1 — Supplementary file containing 4 Tables and 9 Figures about details of IL37 structures, MD systems, FoldX and molecular dynamics simulations. [file mmc1.pdf]

Supporting Information

Rational Design of Monomeric IL37 Variants Guided by Stability and Dynamical Analyses of IL37 Dimers

Inci Sardag[1], Zeynep Sevval Duvenci[2], Serkan Belkaya[3], and Emel Timucin[4]

[1]Bosphorus University, Department of Molecular Biology and Genetics, Istanbul 34342, Turkey
[2]Acibadem University, Department of Biostatistics and Bioinformatics, Institute of Health Sciences, Istanbul 34752, Turkey
[3]Bilkent Ihsan Dogramaci University, Department of Molecular Biology and Genetics, Ankara 06800, Turkey
[4]Acibadem University, Biostatistics and Bioinformatics, School of Medicine, Istanbul 34752, Turkey

List of Tables

S1 SASA measurements of the static structures. . . . 3
S2 MD system details. . . . 3
S3 Protonated residues at pH 2.5. . . . 3
S4 Human variations of IL37 4

List of Figures

S1 (a) Panel displays the AF2 computed homodimer structure of human IL37 colored according to plDDT (yellow: plDDT<60, green: plDDT<70, cyan: plDDT<80, blue: plDDT<90). (b) shows predicted aligned error (PAE) heatmap of the dimer structure. A and B represent dimer subunits. (c) shows superimposition of the AF2 computed dimer and 5hn1. . . . 4
S2 Superimposed structures of 5hn1 and 6ncu chain A-to-chain A and chain A-to-chain B are displayed. . . . 4
S3 (a) Displays the dimer interface and the intramolecular hydrophobic cluster between monomers including Y85 and I86 (b) shows the same residues in (a) on the 6ncu dimer. . . . 5
S4 Pairwise RMSD plots were plotted for the Cα atoms of (a) the dimer wherein the reference structure was dimer, (b) the chain A and B wherein the reference structure was chain A. . . . 5
S5 Score plots for the first three pcs and scree plot for the explained variance were shown for each simulation. RWB color scale indicates the simulation time. . . . 6
S6 *In silico* alanine scanning results were plotted for each IL37 position. Average change in monomer stability ($\Delta\Delta G_{monomer}$) upon alanine mutation was computed by FoldX using seven snapshots including the PDB structures and those extracted from each MD trajectory (5hn1: blue, 6ncu: red, AF2: green). Two insets show close-up views of the region corresponding to the dimer interface of 5hn1. . . . 7
S7 Panel shows the frame range of wild-type and mutant structures selected to perform MM-PBSA binding free energy calculation. For wild-type dimers, 5hn1, AF2 and 6ncu 25 frames were selected both from the initial and final 500 frames (50 ns) of the production simulations. Similar principle was followed for the quadruple mutants by selecting 25 frames from the first and last 10 ns just before dissociation of the dimer interface. . . . 7
S8 Average change in dimer stability ($\Delta\Delta G_{dimer}$) upon site-saturation mutagenesis were calculated by FoldX similarly using seven structures. Residues that were found at or closely located to the dimer interface were selected from both chains. Y-axis shows the selected residues and chain IDs. Diverging color scaling from red to blue represents destabilizing-to-stabilizing substitutions. . . . 8

S9 Average change in monomer stability ($\Delta\Delta G_{monomer}$) calculated by FoldX similarly using seven snapshots. Positions were selected based on their stabilizing impact in alanine scanning analysis (Fig.S6) and/or being found at the dimer interface. Site-saturation mutagenesis are performed for each position. Diverging color scaling from red to blue indicates destabilizing-to-stabilizing substitutions. 8

1 Supplemantary Tables

Table S1 SASA measurements of the static structures.

	5hn1 (PDB)	6ncu (PDB)	AF2
Dimer	175 (167)	168 (172)	171
Chain A	91 (89)	84 (86)	89
Chain B	94 (88)	88 (89)	89
Interface	10 (11)	3 (4)	7

Values in parenthesis indicate SASA (nm^2) after modeling of the missing regions.

Table S2 MD system details.

system	atom	protein	Na^{2+}	Cl^-	water	x	y	z
5hn1	114606	4985	104	116	36467	108	108	108
6ncu	108672	4870	98	108	34532	106	106	106
AF2	105572	4996	95	107	33458	105	105	105

Dimensions (x, y, z) of final MD systems are in Angstrom units.

Table S3 Protonated residues at pH 2.5.

systems	aa	chain A	chain B
5hn1	D	64, 66, 73, 82, 123, 191	64, 66, 73, 82, 123, 125, 191
	E	89, 117, 138, 148, 168, 185, 194, 200	89, 104, 117, 138, 148, 168, 185, 194, 200
	H	63, 67, 130, 172, 198	63, 67, 130, 172, 198
6ncu	D	64, 66, 73, 82, 123, 125, 191	64, 66, 73, 82, 123, 125, 191
	E	89, 104, 117, 138, 148, 168, 185, 194, 200	89, 104, 117, 138, 148, 168, 185, 194, 200
	H	63, 67, 130, 172, 198	63, 67, 130, 172, 198

Table S4 Human variations of IL37

See TableS4.csv

2 Supplementary Figures

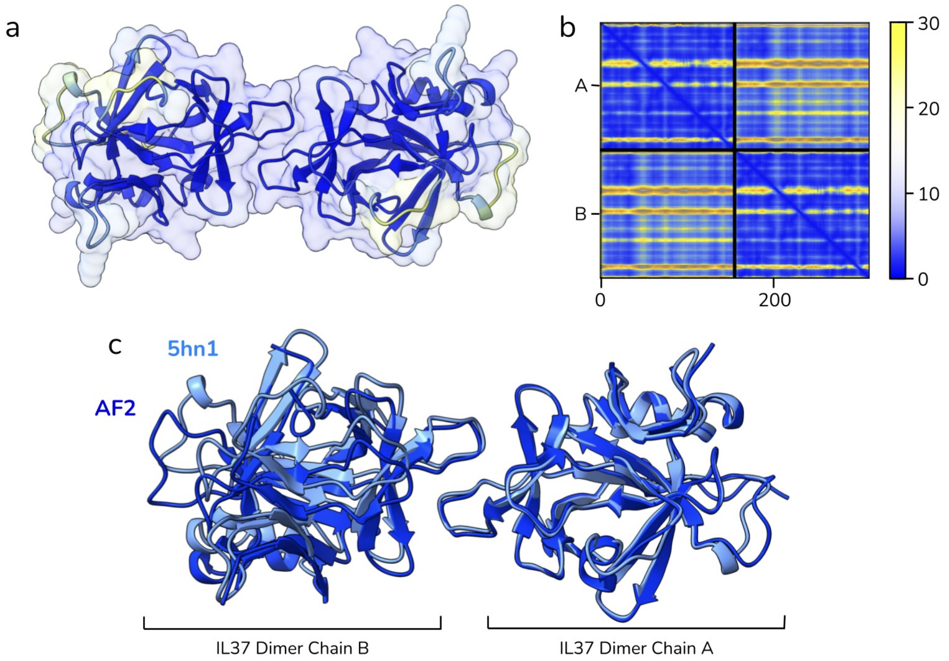

Figure S1 (a) Panel displays the AF2 computed homodimer structure of human IL37 colored according to plDDT (yellow: plDDT<60, green: plDDT<70, cyan: plDDT<80, blue: plDDT<90). (b) shows predicted aligned error (PAE) heatmap of the dimer structure. A and B represent dimer subunits. (c) shows superimposition of the AF2 computed dimer and 5hn1.

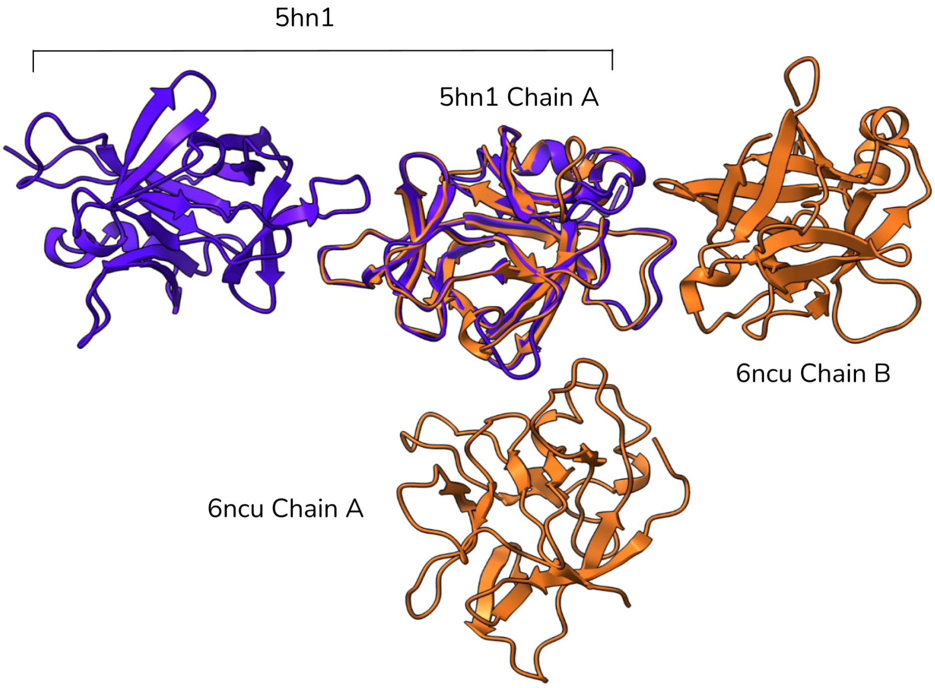

Figure S2 Superimposed structures of 5hn1 and 6ncu chain A-to-chain A and chain A-to-chain B are displayed.

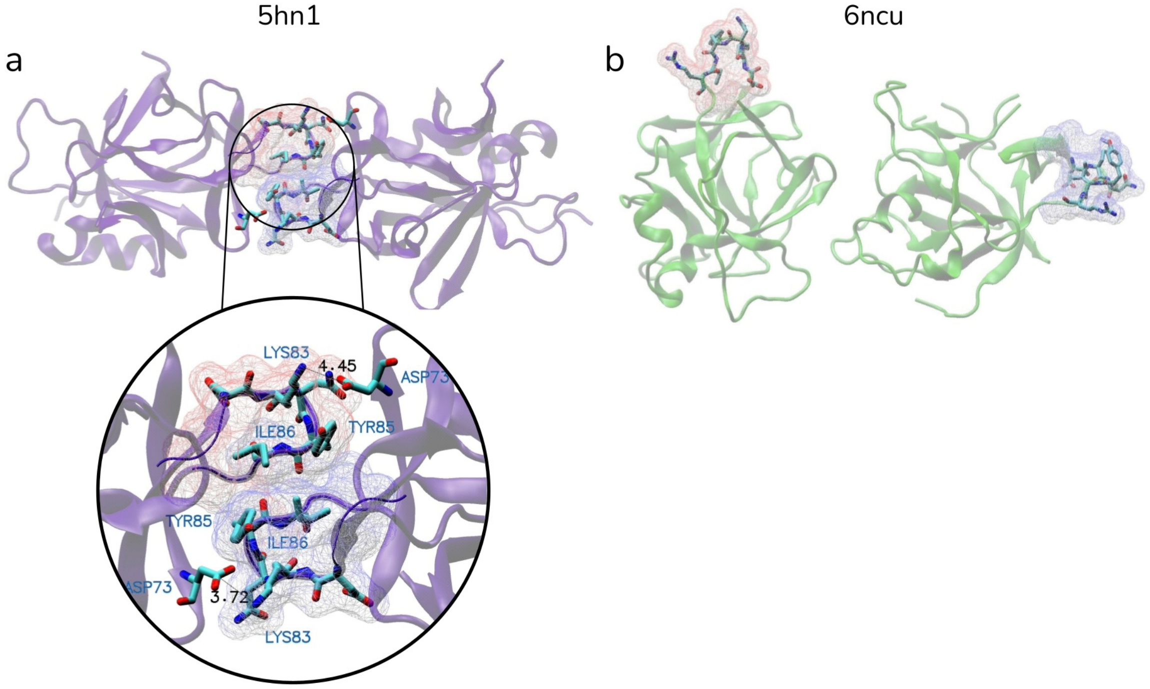

Figure S3 (a) Displays the dimer interface and the intramolecular hydrophobic cluster between monomers including Y85 and I86 (b) shows the same residues in (a) on the 6ncu dimer.

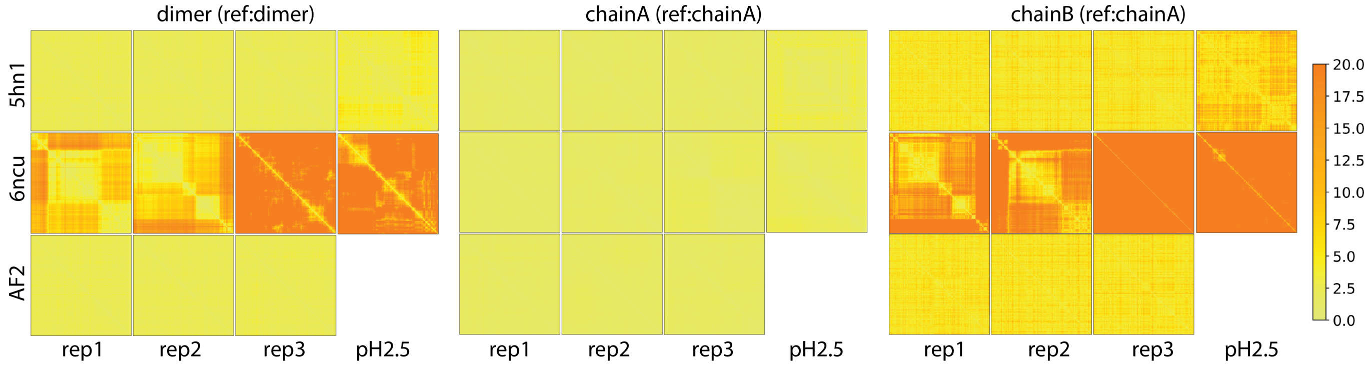

Figure S4 Pairwise RMSD plots were plotted for the Cα atoms of (a) the dimer wherein the reference structure was dimer, (b) the chain A and B wherein the reference structure was chain A.

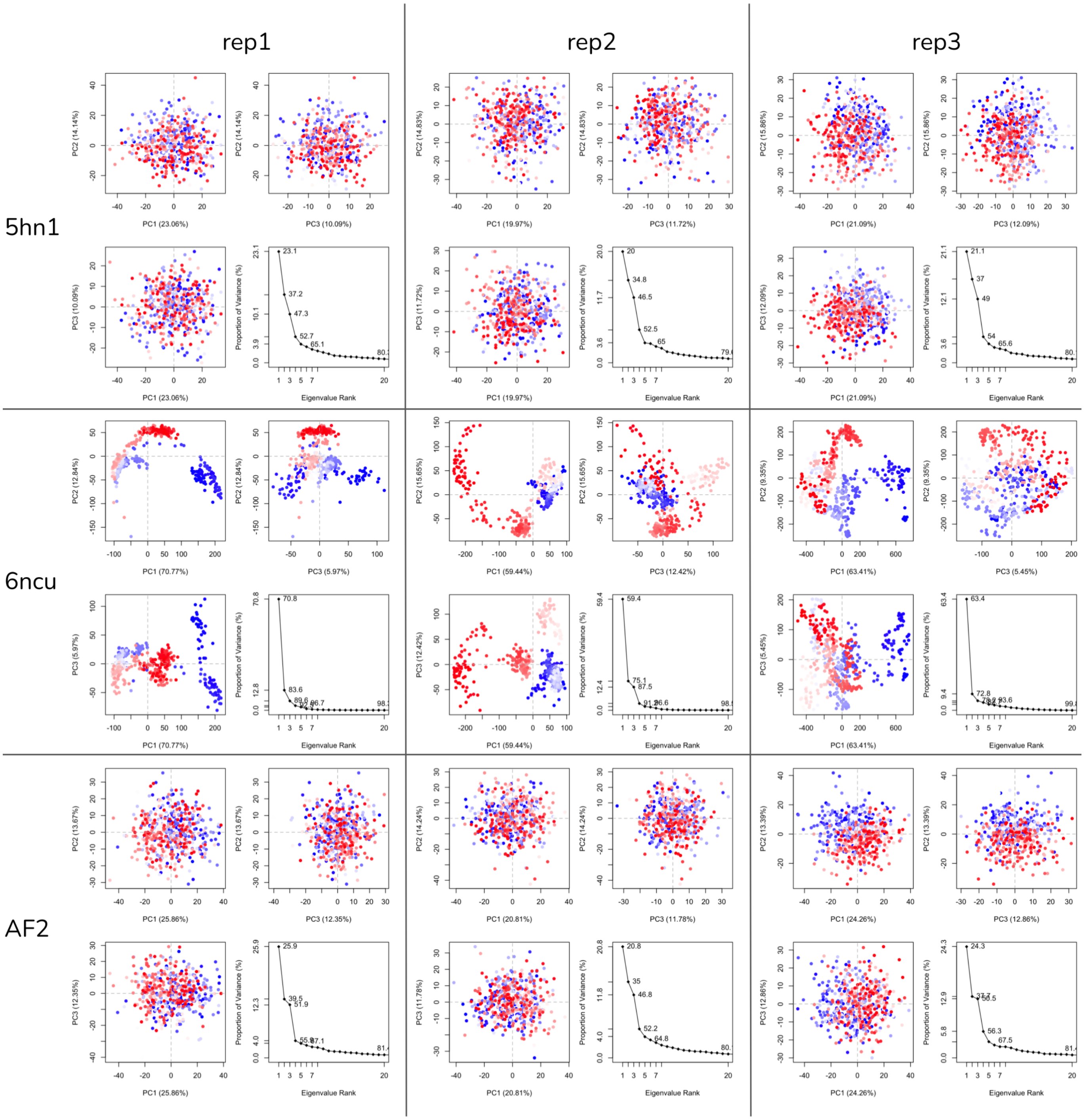

Figure S5 Score plots for the first three pcs and scree plot for the explained variance were shown for each simulation. RWB color scale indicates the simulation time.

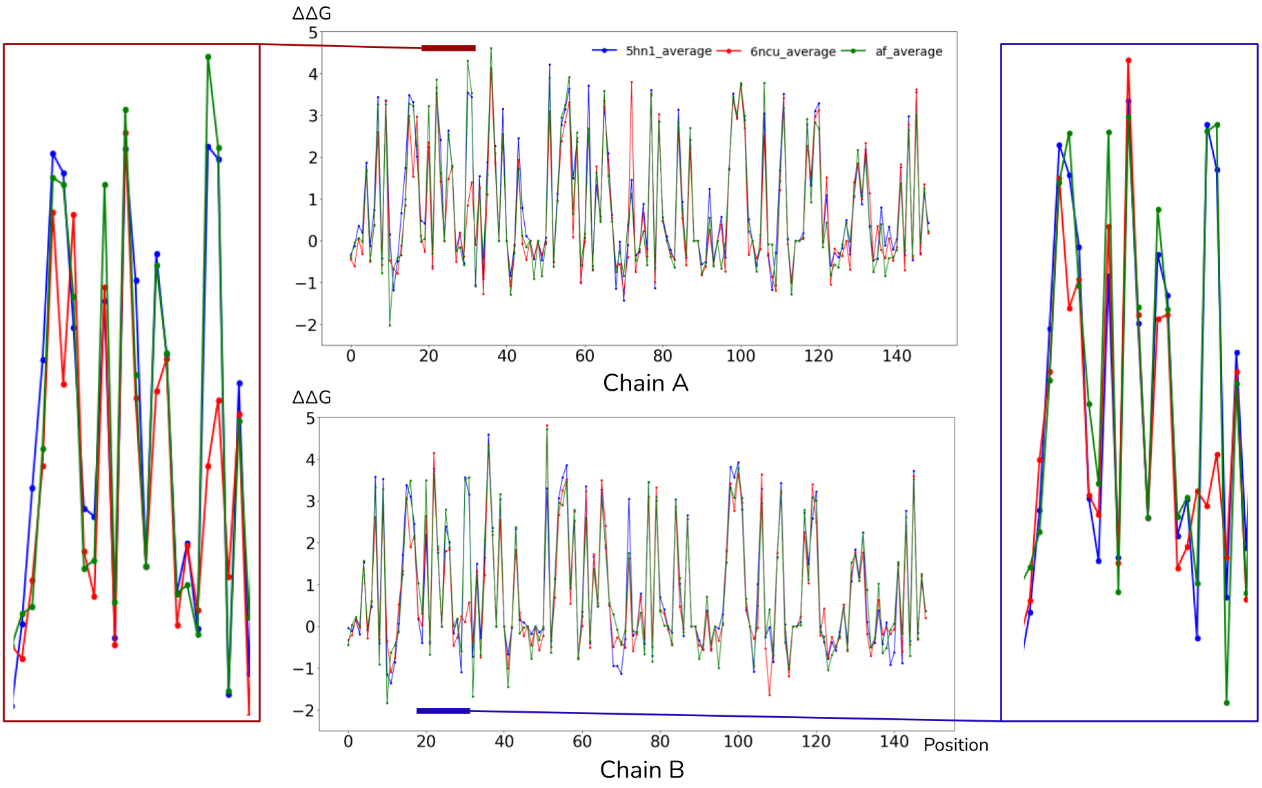

Figure S6 *In silico* alanine scanning results were plotted for each IL37 position. Average change in monomer stability ($\Delta\Delta G_{monomer}$) upon alanine mutation was computed by FoldX using seven snapshots including the PDB structures and those extracted from each MD trajectory (5hn1: blue, 6ncu: red, AF2: green). Two insets show close-up views of the region corresponding to the dimer interface of 5hn1.

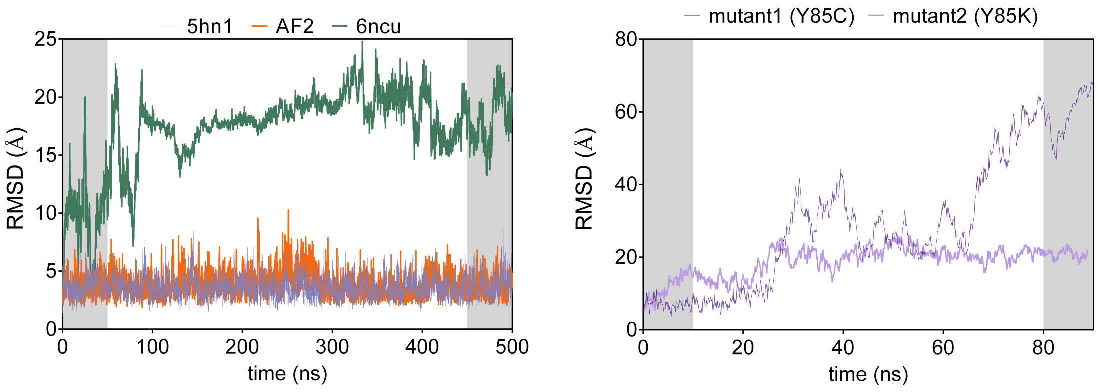

Figure S7 Panel shows the frame range of wild-type and mutant structures selected to perform MM-PBSA binding free energy calculation. For wild-type dimers, 5hn1, AF2 and 6ncu 25 frames were selected both from the initial and final 500 frames (50 ns) of the production simulations. Similar principle was followed for the quadruple mutants by selecting 25 frames from the first and last 10 ns just before dissociation of the dimer interface.

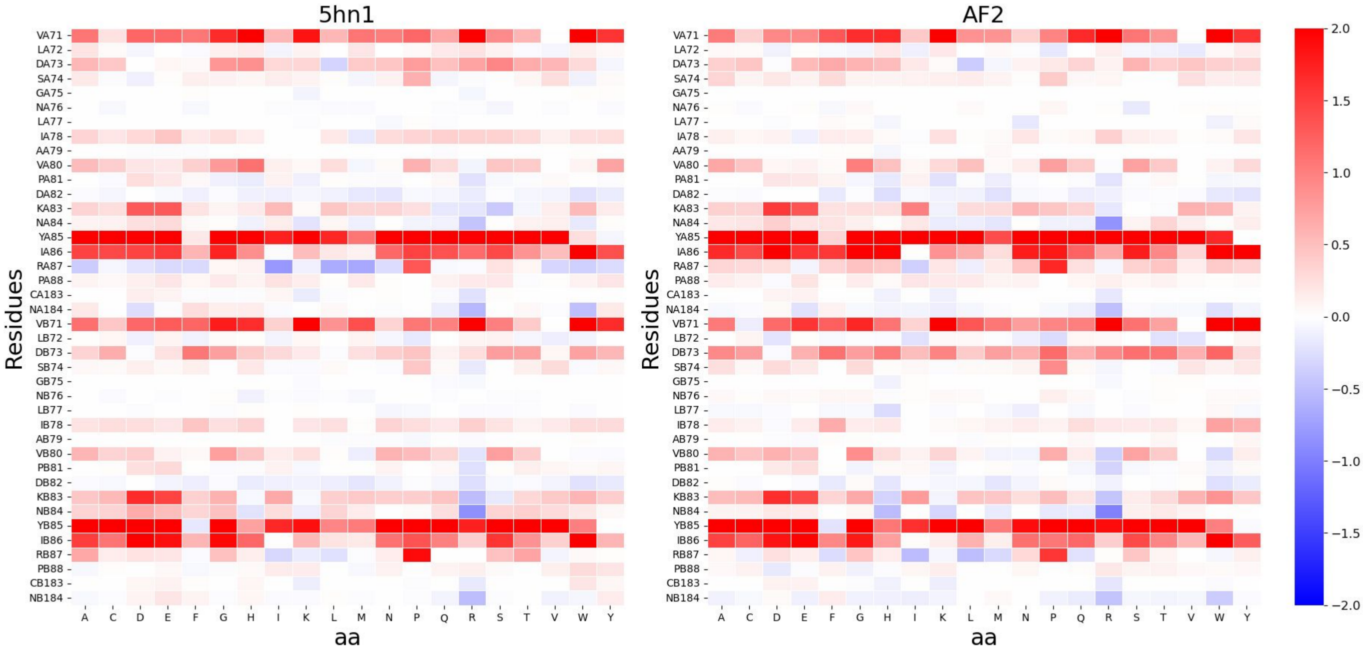

Figure S8 Average change in dimer stability ($\Delta\Delta G_{dimer}$) upon site-saturation mutagenesis were calculated by FoldX similarly using seven structures. Residues that were found at or closely located to the dimer interface were selected from both chains. Y-axis shows the selected residues and chain IDs. Diverging color scaling from red to blue represents destabilizing-to-stabilizing substitutions.

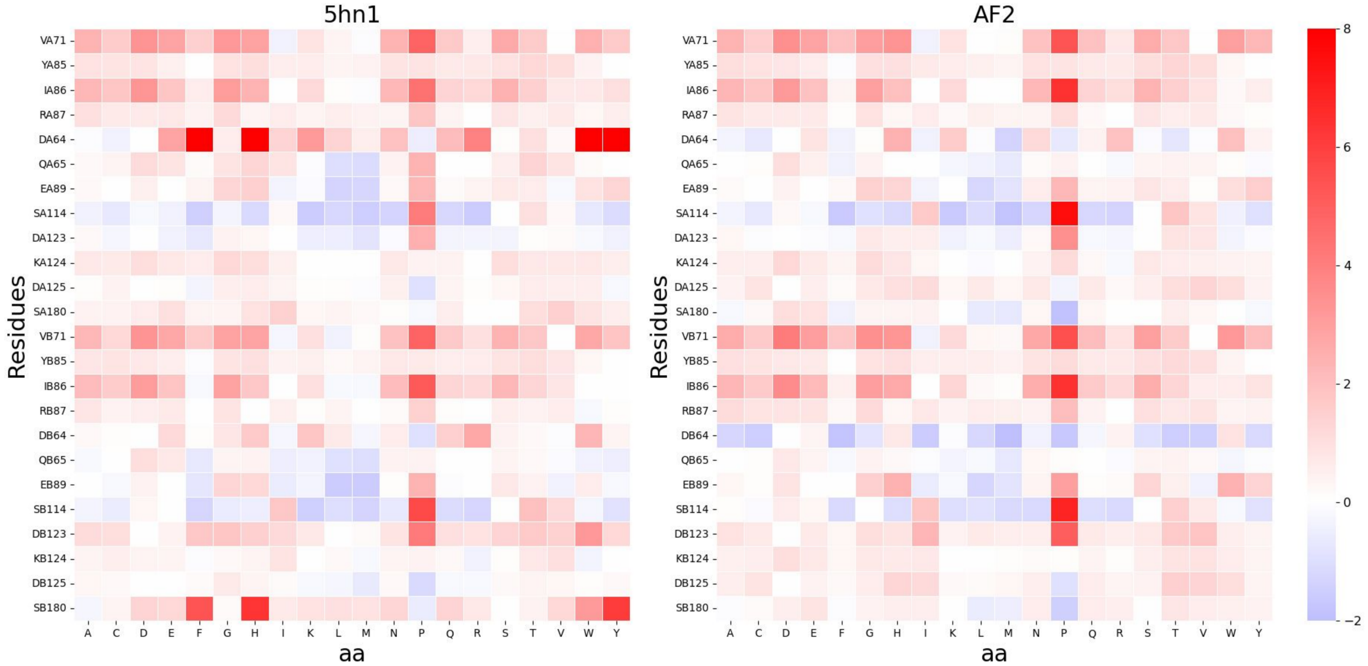

Figure S9 Average change in monomer stability ($\Delta\Delta G_{monomer}$) calculated by FoldX similarly using seven snapshots. Positions were selected based on their stabilizing impact in alanine scanning analysis (Fig.S6) and/or being found at the dimer interface. Site-saturation mutagenesis are performed for each position. Diverging color scaling from red to blue indicates destabilizing-to-stabilizing substitutions.
